# Up-regulated CD38 by daphnetin alleviates lipopolysaccharide-induced lung injury via inhibiting MAPK/NF-κB/NLRP3 pathway

**DOI:** 10.1186/s12964-023-01041-3

**Published:** 2023-03-30

**Authors:** Yujie Guo, Huiqing Zhang, Zhe Lv, Yuna Du, Dan Li, Hui Fang, Jing You, Lijun Yu, Rong Li

**Affiliations:** 1 Department of Clinical Laboratory, Jiangxi Provincial People’s Hospital and The First Affiliated Hospital of Nanchang Medical College, Nanchang, China; 2 Department of Medical Microbiology and Immunology, School of Basic Medical Sciences, Nanchang University, Nanchang, China

**Keywords:** Daphnetin, Sepsis, Lung injury, CD38, Inflammation, Pyroptosis

## Abstract

**Background:**

Sepsis is a life-threatening organ dysfunction syndrome resulted from severe infection with high morbidity and mortality. Cluster of differentiation 38 (CD38) is a multifunctional type II transmembrane glycoprotein widely expressed on the surface of various immunocytes membranes that mediates host immune response to infection and plays an important role in many inflammatory diseases. Daphnetin (Daph), isolated from the daphne genus plant, is a natural coumarin derivative that possesses anti-inflammatory and anti-apoptotic effects. The current study aimed to investigate the role and mechanism of Daph in alleviating lipopolysaccharide (LPS)-induced septic lung injury, and to explore whether the protective effect of Daph in mice and cell models was related to CD38.

**Methods:**

Firstly, network pharmacology analysis of Daph was performed. Secondly, LPS-induced septic lung injury in mice were treated with Daph or vehicle control respectively and then assessed for survival, pulmonary inflammation and pathological changes. Lastly, Mouse lung epithelial cells (MLE-12 cells) were transfected with CD38 shRNA plasmid or CD38 overexpressed plasmid, followed by LPS and Daph treatment. Cells were assessed for viability and transfection efficiency, inflammatory and signaling.

**Results:**

Our results indicated that Daph treatment improved survival rate and alleviated pulmonary pathological damage of the sepsis mice, as well as reduced the excessive release of pro-inflammatory cytokines IL-1β, IL-18, IL-6, iNOS and chemokines MCP-1 regulated by MAPK/NF-κB pathway in pulmonary injury. Daph treatment decreased Caspase-3 and Bax, increased Bcl-2, inhibited nucleotide-binding domain (NOD)-like receptor protein 3 (NLRP3) inflammasome‐mediated pyroptosis in lung tissues of septic lung injury. Also, Daph treatment reduced the level of excessive inflammatory mediators, inhibited apoptosis and pyroptosis in MLE-12 cells. It is noteworthy that the protective effect of Daph on MLE-12 cells damage and death was assisted by the enhanced expression of CD38.

**Conclusions:**

Our results demonstrated that Daph offered a beneficial therapeutic effect for septic lung injury via the up-regulation of CD38 and inhibition of MAPK/NF-κB/NLRP3 pathway.

Video Abstract

**Supplementary Information:**

The online version contains supplementary material available at 10.1186/s12964-023-01041-3.

## Background

Sepsis is one of the most fatal diseases worldwide, characterized by multiple organ failure, mainly due to uncontrolled inflammatory response [1]. Sepsis is often associated with organ dysfunction induced by dysregulation of host defense against infection, and the lung is the most vulnerable organ during sepsis [2, 3]. Serious lung injury may lead to acute lung injury/acute respiratory distress syndrome ((ALI/ARDS) and even respiratory failure [4]. There are reports showed that LPS is commonly used to establish ALI models in which LPS is the primary endotoxin of gram-negative bacteria, stimulating the expression of inflammatory cytokines by binding toll-like Receptor 4 (TLR4), triggering an acute inflammation reaction and accelerating cell infiltration in lung tissues [5–7]. Accordingly, inhibition of inflammatory response may be a potential way to prevent ALI.

Abundant previous reports have showed that multiply signal pathways play an essential role in the regulation of inflammatory response during ALI, including mitogen-activated protein kinases (MAPKs), nuclear factor-kappa B (NF-κB) and nucleotide-binding domain (NOD)-like receptor protein 3 (NLRP3) inflammasome [8, 9]. On the one hand, MAPKs family, which is composed of extracellular signal-regulated kinase1/2 (ERK1/2), p38 kinase and c-Jun NH2-terminal kinase (JNK) activated by LPS and regulate the transcription of inflammatory cytokines such as inducible nitric oxide synthase (iNOS) and cyclooxygenase-2(COX-2) [10]. Moreover, NF-κB, which comprises the p50/p65 and the inhibitor of κB (IκB) protein, is essential for host defense and also mediates these pro-inflammatory mediators and cytokines production and secretion [11]. On the other hand, NLRP3 is a multiprotein complex, including NOD-like receptor (NLR), the adaptor protein apoptosis-associated speck-like protein containing a caspase-recruitment domain (ASC) and Caspase-1 [12]. Once initiated by stimuli, for instance LPS, NLRP3 proteins polymerize and bind to the ASC adaptor, which in turn promotes the recruitment and activation of Pro-caspase-1 and mature form of IL-1β, IL-18, leading to inflammatory response [13, 14]. At the same time, activated Caspase-1 can cleave gasdermin-D (GSDMD) and induce pro-inflammatory cell death called pyroptosis. More and more studies have shown that NLRP3-meditated pyroptosis may cause vital injury in different organs affected by sepsis [15–17]. Hence, the inhibition of MAPKs and NF-κB signaling pathway, NLRP3 inflammasome activation may contribute to reducing inflammation for the amelioration of ALI.

Cluster of differentiation 38 (CD38), a 46-kDa type II transmembrane glycoprotein, which is composed of 300 amino acids encoded by homologous genes located on chromosome 4 and 5 in humans and mice respectively [18]. CD38 was initially recognized as ADP-ribosyl cyclase (ADPR-cyclase) and cyclic ADP-ribose hydrolase (cADPR-hydrolase), as well as a key nicotinamide adenine dinucleotide (NAD^+^)-dependent enzyme in mammalian tissues [19, 20]. CD38 is widely expressed in various immunocytes membranes and involved in the natural immune response against infection [21]. Accumulated evidence indicates that CD38 plays an important role in many inflammatory diseases, such as lung injury associated with sepsis [22, 23]. Recently, it was demonstrated that CD38 deficient mice increased the expression of IL-1β and MCP-1 and aggravated lung injury through TLR4/ERK/NF-κB pathway in sepsis [23]. And studies have been reported that blocking CD38 pathway can protect hippocampal cells from apoptosis, oxidative stress and ultrastructural damage in sepsis rats [24]. Therefore, further experimental studies are needed to confirm the role of CD38 in the immunosuppressive phase of sepsis and whether selective interference of CD38 activity can improve the immunopathological changes caused by excessive inflammation.

**Fig. 1:**
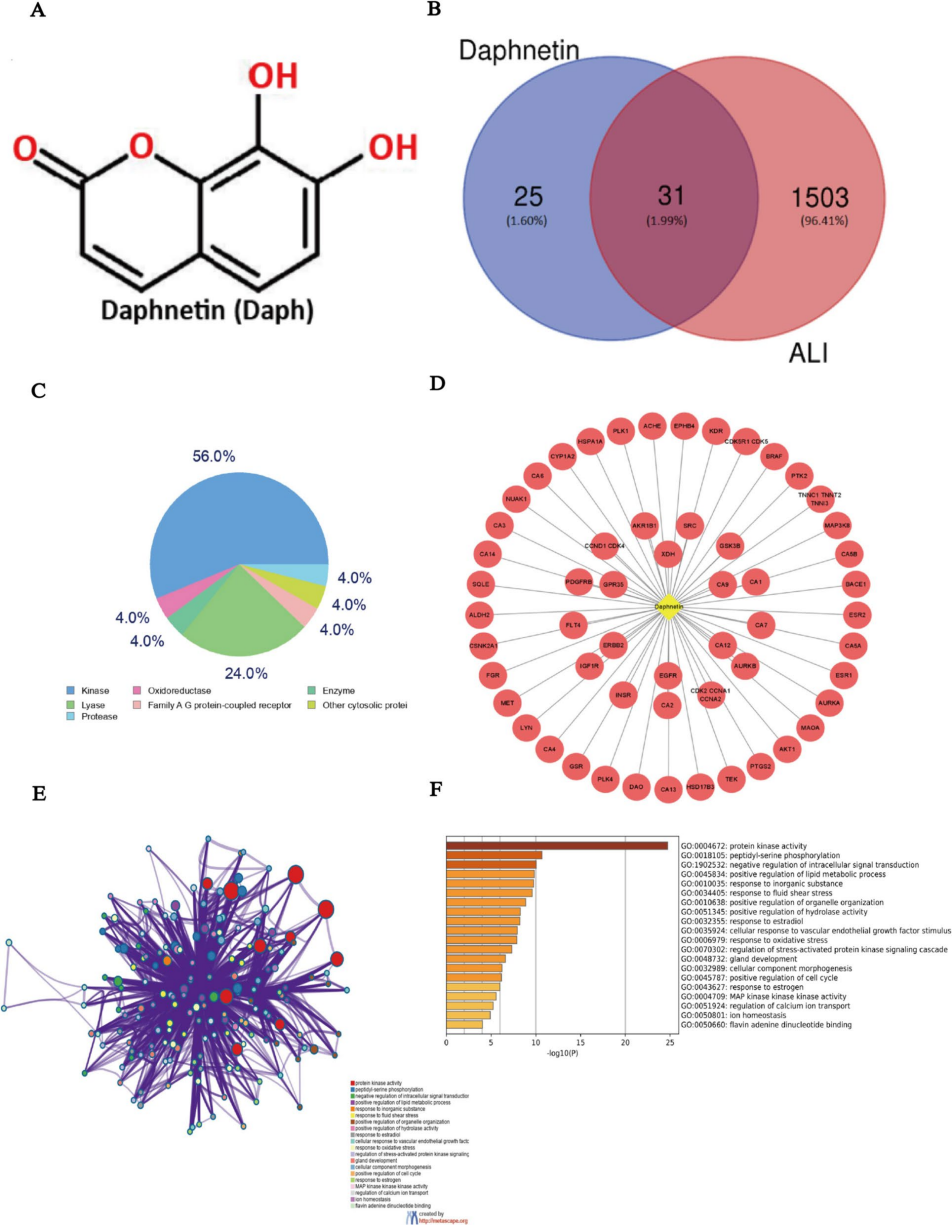
Network pharmacology analysis of Daphnetin based on ALI. **a** The chemical structure of Daphnetin. **b** The common target genes of Daphnetin and ALI. **c** The target classes of Daphnetin. **d** The potential targets of Daphnetin in the treatment of ALI were identified using the Swiss Target Prediction. **e–f** GO annotation and KEGG were used to analyze these target genes

There are numerous compounds that exert anti-inflammatory potential through the inactivation of MAPK/NF-κB pathway and NLRP3 inflammasome [8, 25, 26]. Daphnetin (7,8-dithydroxycoumarin, Daph; Fig. 1a), a coumarin derivative from daphne genus plant, is the primary component of Chinese herb medicine Zushima, which is widely used in clinical treatment of various inflammatory diseases [27]. Furthermore, it was proven that Daph could effectively reduce the level of inflammatory factors IL-6 and TNF-α by inhibiting the activation of NF-κB signaling pathway, which has a protective effect against LPS-induced septic lung injury in mice [28]. However, whether MAPK and NLRP3 inflammasome restricted by Daph and the resulting protection against LPS-induced lung injury is rarely reported. Thus in the present study, we proposed to systematically investigate the effects and the underlying mechanisms of Daph in LPS-induced lung injury and inflammatory response in both mouse and cell model. Additionally, we discussed whether CD38, regulated by Daph, could mediate the inhibition of MAPK/NF-κB pathway and NLRP3 inflammasome.

## Materials and methods

### Network pharmacology analysis

Pharmaceutical active ingredient of Daph was assessed by Traditional Chinese Medicine Systems Pharmacology database (TCMSP, https://tcmspw.com/), and the potential targets of each active ingredient were identified through using the Swiss Target Prediction (http://www.swisstargetprediction.ch/). Additionally, gene ontology (GO) and Kyoto Encyclopedia of Genes and Genomes (KEGG) analysis were established by using Metascape platform (https://metascape.org/). Functional modules of Drug-target-pathway were constructed through protein protein interaction (PPI) via using Cytoscape (http://cytoscape.org/).

### Chemicals and antibodies

Daphnetin (Daph) with purity ≥ 98% was provided by Herbpurify CO., LTD (Chengdu, China). LPS (O111:B4) was purchased from Sigma-Aldrich (USA). Dimethyl sulfoxide (DMSO) was obtained from Solarbio Technology (Beijing, China). Primary antibodies were listed in Additional file 1: Table S1.

### Animals and models

Male, healthy, wild-type (WT) C57BL/6J mice (4 weeks old, specific pathogen free) were purchased from the Laboratory Animal Center of Wuhan University. All groups of mice were raised in sterile filter-top cages with a 12-h light–dark cycle in SPF Animal Facility at Laboratory animal center of Nanchang University and kept well fed and watered. Eight-week-old male mice (22 ± 2 g) were selected for the experiment. All experiments were conducted in accordance with the guidelines specified by the Animal Care and Usage Committee of Nanchang University.

Twelve male mice were randomly divided into four groups, and the sepsis model was established by intraperitoneal injection of LPS at different concentrations (0, 5, 10, 20 mg/kg). As performed in previous studies [29], lung tissues of mice were harvested 3 h later and the expression of inflammatory cytokines in each group were measured, so as to determine the most appropriate concentration of LPS for sepsis model. To investigate the therapeutic effect of Daph on sepsis mice, the mice were randomly distributed into five groups (n = 10/group for protocol 1 and n = 3/group for protocol 2): the control (PBS) group, the 0.1% DMSO group, the Daph (5 mg/kg dissolved in 0.1% DMSO) group and the LPS (10 mg/kg) + Daph (5 mg/kg) group. The mice subjected to protocol 1 were received Daph (5 mg/kg) and LPS (10 mg/kg) by intraperitoneal injection. Survival was monitored every half hour up to 3 days. In addition, the mice subjected to protocol 2 were administered Daph (5 mg/kg) for 2 h after 3 h of LPS treatment and then euthanized. Lung tissues were harvested and used for hematoxylin and eosin (H&E) staining, real-time quantitative polymerase chain reaction (RT-qPCR) and Western blot assay.

### Hematoxylin and eosin staining

After experiment, lung tissues were fixed in 4% paraformaldehyde solution overnight, embedded in paraffin, and then sectioned at 3-μm thickness using a microtome. After deparaffinization and rehydration, lung sections were stained with hematoxylin and eosin for microscopic examination. The sections were viewed by electron microscope at magnifications of 20 × or 40 × , and three fields were randomly selected for each section by a pathologist using a double-blind method. Histopathological changes were evaluated by the following four indicators:1. Intrapulmonary hemorrhage; 2. Formation of edema fluid; 3. infiltration of inflammatory cells in the lung; 4. Formation of hyaline membrane (thickened alveolar wall). The scoring standard of lung injury was: 0 score, normal; 1 score, very mild impairment (< 25% field area); 2 scores, mild impairment (25% to 50% of visual field area); 3 scores, moderate impairment (50% to 75% of visual field area); 4 scores, severe impairment (> 75% view area).

### Cell culture and viability evaluation

Mouse lung epithelial cells line (MLE-12 cells) were purchased from the Shanghai Institute of Biochemistry and Cell Biology, Chinese Academy of Sciences. Cells were incubated in high glucose Dulbecco’s Modified Eagle’s Medium (DMEM, Solarbio Technology, Beijing, China) containing 4500 mg/L glucose, and supplemented with 10% fetal bovine serum (FBS, Invitrogen-Gibco, USA), 1% penicillin and streptomycin at 37 °C in a thermostatic incubator containing 5% CO2. Cells in the logarithmic growth phase with stable state were selected for further experiments.

Cell viability was determined using CCK-8 Cell Viability Assay Kit (Fude Biotech, Hangzhou, China). MLE-12 cells were treated with various concentrations of Daph or LPS for 24 h respectively, and then cells were incubated with CCK-8 solution at 37 °C for 2–4 h. The absorbance was detected at 450 nm using a microplate reader (Bio-Tek, Vermont, USA).

### Cell treatment

Cells (2 × 10^5^ cells/well) were seeded into 6-well plates and cultured for 24 h until reaching approximately 70% confluence. To evaluate the role of inflammation, MLE-12 cells were treated with different concentrations of LPS (0, 1, 2.5, 5, 10, 20 μg/ml) for 24 h. To determine the effect of Daph, MLE-12 cells were treated with LPS for 12 h, and then co-treated with different concentrations of Daph (5, 10, 20 μg/ml) the following 12 h. To verify the effect of Daph on CD38 expression, MLE-12 cells were treated with different concentrations of Daph (0, 5, 10, 20 μg/ml) for 24 h.

In addition, MLE-12 cells were transfected with CD38 shRNA plasmid (RiboBio co., LTD, Guangzhou, China) or CD38 overexpression plasmid (Vegen Biotech, Zhenjiang, China) using Lipofectamine 2000™ reagent (Invitrogen, Carlsbad, CA, USA) for 24 h, followed by exposure to LPS and Daph treatment. After 48 h, the protein expression of CD38 was detected by western blot to verify transfection efficiency. The sequences of CD38 shRNA plasmid and CD38 overexpressed plasmid were listed in Additional file 2: Table S2.

### RNA extraction and real-time quantitative PCR assay

Total RNA was extracted from lung tissues and MLE-12 cells by using TRIzol Reagent (Ambion RNA™, Life technologies^®^). All procedures were carried out according to the manufacture’s guidelines. And then, the Transcription first-strand cDNA Synthesis Kit (Takara Biotech, Japan) was used to synthesize RNA (2 μg) into cDNA. The RT-qPCR was performed using SYBR^®^ Premix Ex Taq™II (Takara Biotech, Japan) and the StepOne™ PLUS Real-Time PCR System (Applied Biosystems, Carlsbad^®^, CA, USA). The sequences of all primers are listed in Additional file 2: Table S2. We detected the threshold cycle (Ct) for all genes and determined their relative expression levels compared to GAPDH. Experiments were repeated 3 times.

### Immunohistochemical staining

The expression of NF-κB p65, ERK1/2, JNK in lung tissues was evaluated by immunohistochemistry according to the standard protocol. In brief, the paraffin sections of pulmonary samples were heated in in an oven at 65 °C overnight, deparaffinized in xylene for 5 min three times, rehydrated by graded ethanol solutions, and blocked by incubation in 0.3% fresh hydrogen peroxide for 10 min. The sections were blocked with 3% bovine serum albumin (BSA) at 25 °C and incubated with anti-NF-κB p65, anti-ERK1/2, anti-JNK primary antibody for 50 min at room temperature, and then incubated with biotin-conjugated secondary antibodies for 25 min at room temperature after a thorough wash in PBS three times. Then, the sections were incubated with chromogenic agent diaminobenzidine (DAB) and re-stained with hematoxylin. After dehydrating and drying, the sections were fixed on glass slides and observed under the microscope.

### Western blot analysis

The lung tissues or MLE-12 cells were ground up and lysed in the RIPA Lysis Buffer (Solarbio Technology, Beijing, China) with a protease inhibitor PMSF (100:1) to extract protein, and protein concentration was determined by BCA Protein Assay kit. The equivalent protein was separated into 10% SDS-PAGE gel, transferred to polyvinylidene fluoride (PVDF) membranes and blocked with 5% fat-free milk in TBST. The membranes were washed by TBST and then incubated with primary antibodies overnight at 4^◦^C. Then, the membranes were washed and incubated with horseradish peroxidase- (HRP-) conjugated anti-rabbit or anti-mouse IgG (1:10,000 dilution; Proteintech, USA) for 2 h at room temperature. The protein bands were visualized using the ECL system (Sage creation) according to the manufacturer’s instructions. All the original results of Western blot analysis are listed in Additional file 3: Figure S3.

### Statistical analysis

Each experiment is independent and repeatable. All data were depicted as the means ± standard deviation (SD). A paired t-test was used to determine statistically significant differences between two groups and comparison between multi-groups was assessed by one-way ANOVA with Tukey’s multiple comparison test by GraphPad Prism 5.0(USA). A *p*-value of less than 0.05 was considered to be statistically significant.

## Results

### Network pharmacology analysis of daphnetin

To understand the therapeutic effects of Daph on sepsis-related lung injury, we analyzed the pharmacological effects of Daph through network diagram. Daph is a natural plant-derived product with the chemical structure of 7, 8-dihydroxycoumarin (Fig. 1a). Our results show that Daph has good drug potency for the treatment of acute lung injury (ALI), with 31 supposedly identified target genes (Fig. 1b). The target classes and the potential targets of Daph in the treatment of ALI (Fig. 1c, d). GO annotation and KEGG analysis indicated that these target genes were mainly related to intracellular signal transduction and regulation, inflammatory response and oxidative stress (Fig. 1e–f).

### LPS induced pro-inflammatory cytokines production in lung tissues of mice

**Fig. 2:**
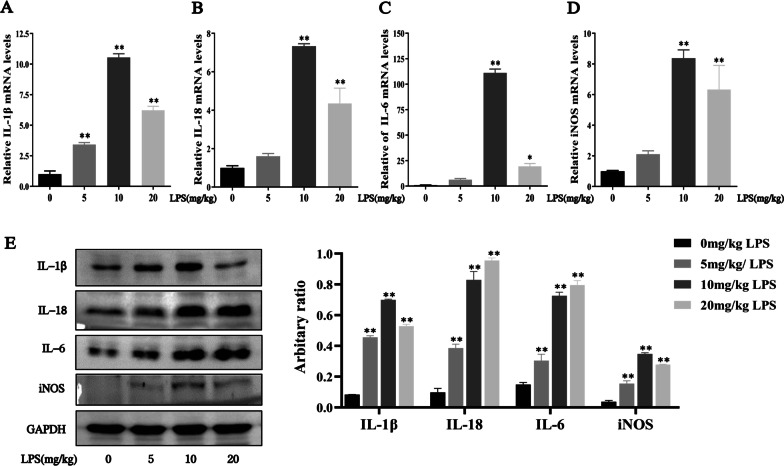
LPS induced sepsis-related lung injury in mice. **a-d** The mRNA expression of IL-1β, IL-18, IL-6 and iNOS in lung tissue were measured by RT-qPCR. **e** The protein expression of IL-1β, IL-18, IL-6 and iNOS in lung tissue were detected by Western blot analysis, and the relative expression of previous protein were quantified by Image J software and GAPDH was acted as an internal control. All results were expressed as the means ± SD of three independent experiments. *P < 0.05, **P < 0.01 versus 0 mg/kg LPS

In order to induce sepsis-related lung injury, we used intraperitoneal injection of LPS at an increasing concentration to establish sepsis mouse model. As shown in Fig. 2a–e, our results showed that the mRNA and protein expression of pro-inflammatory cytokines IL-1β, IL-18, IL-6 and iNOS significantly increased at the concentration of 10 or 20 mg/kg after 3 h of LPS challenged. Thus, LPS induced pulmonary injury in mice and 10 mg/kg LPS was chosen in following study.

### Daph treatment inhibited the expression of pulmonary inflammatory cytokines and chemokines, improved pathological injury of lung tissues and reduced lethality in LPS-induced lung injury in mice

**Fig. 3:**
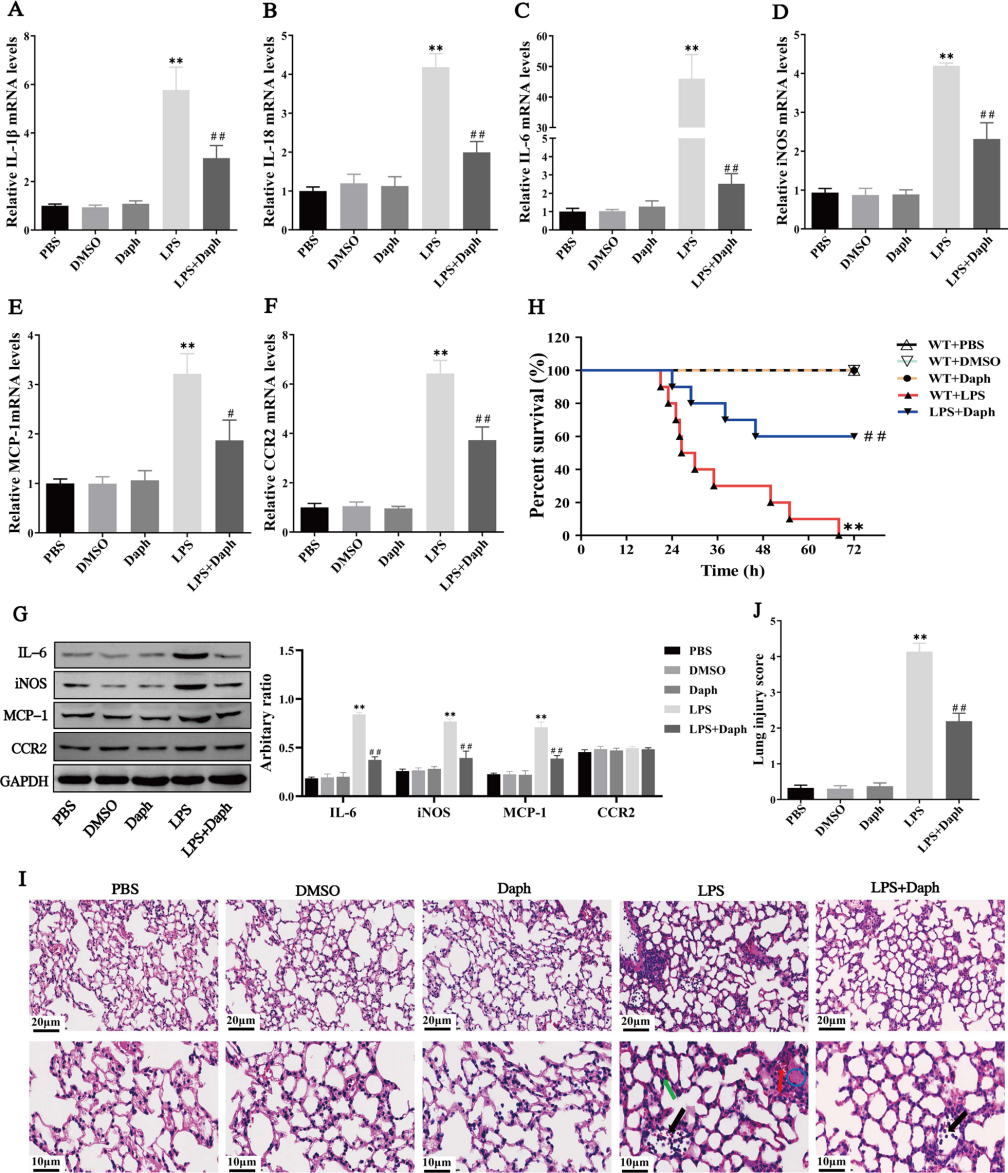
Daph treatment alleviated pulmonary inflammation and reduced mortality in LPS-induced lung injury. **a-f** The mRNA expression of IL-1β, IL-18, IL-6, iNOS, MCP-1 and CCR2 in lung tissue were measured by RT-qPCR. **g** The protein expression of IL-6, iNOS, MCP-1 and CCR2 were detected by Western blot analysis, and relative expression of previous protein were quantified by Image J software and GAPDH was acted as an internal control. **h** The survival rates of the sepsis mice were observed after Daph treatment, WT + PBS, WT + DMSO and WT + Daph were used as the control group among them, so these three lines overlapped together. **i-j** The pathological changes in lung tissue were compared by H&E staining, and the lung injury scores were evaluated. The black arrows indicate inflammatory cell infiltrations, the green arrows indicate thickened of alveolar walls, the red arrows indicate intrapulmonary hemorrhage and the blue cycles indicate formation of edema fluid. All results were expressed as the means ± SD of three independent experiments. **P < 0.01 versus the control group; #*P* < 0.05, ##*P* < 0.01 versus LPS group

To evaluate whether Daph was able to revolve the LPS evoked inflammation storm, we employed RT-qPCR to detect the expression of pro‑inflammatory factors in lung tissues. As shown in Fig. 3a–f, Daph obviously inhibited the gene levels of pro-inflammatory cytokines IL-1β, IL-18, IL-6, iNOS, chemokine MCP-1 and its receptor CCR2 in lung tissues compared with LPS group. The protein expression of these pro-inflammatory cytokines and chemokines were remarkedly suppressed by Daph, while CCR2 showed no significantly difference (Fig. 3g). To investigate the effect of Daph on the survival rate of sepsis mice, mice were treated with Daph after intraperitoneal injection of LPS or vehicle for 3 h, and the survival of mice was observed. Survival curve result showed that the mice died at 21 h after LPS challenged, and survival rate reached 0 at 68 h, while treatment with Daph effectively increased survival rate up to 60% (Fig. 3h). Histopathological examination revealed that the lung tissues structure of mice in LPS group exhibited a thickened alveolar septum, intrapulmonary hemorrhage and formation of edema fluid, and marked inflammatory cell infiltration compared with the control group, whereas the mice treated with Daph exhibited lessened pathological lesions characterized by reduced interstitial edema, less inflammatory cell infiltration in lung tissues (Fig. 3i). Lung histological scores dramatically increased by LPS stimulation, while treatment with Daph significantly reduced the corresponding scores (Fig. 3j). Therefore, Daph treatment could reduce the mortality, alleviate pathological damage in lung tissues, and decrease inflammatory cytokines and chemokines expression in LPS-induced lung injury in mice.

### Daph suppressed TLR4-NF-κB/MAPK pathway in the lungs of septic mice

**Fig. 4:**
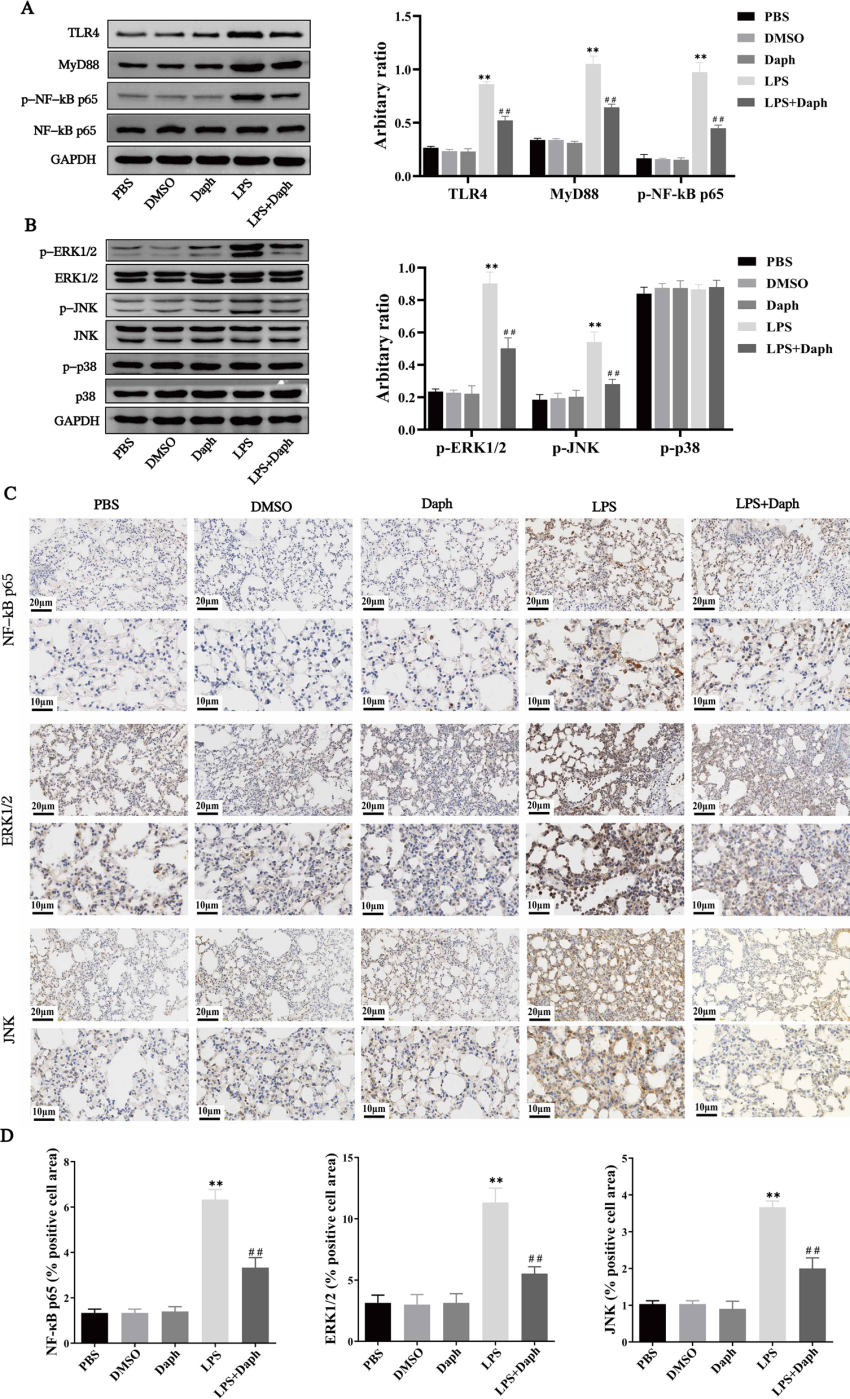
Effects of Daph on TLR4-NF-κB/MAPK signaling pathway activation in LPS-induced lung injury. **a-b** The protein expression of TLR4, MyD88, p-NF-κB p65, NF-κB p65, p-ERK1/2, ERK1/2, p-JNK, JNK, p-p38 and p38 in lung tissue were detected by Western blot analysis, and relative expression of previous protein were quantified by Image J software and GAPDH was acted as an internal control. **c** The distribution of NF-κB p65, ERK1/2 and JNK in lung tissue were detected by Immunohistochemistry staining. **d** The calculated NF-κB p65, ERK1/2, JNK-positive cells (brown area) in lung tissue for all groups. All results were expressed as the means ± SD of three independent experiments. **P < 0.01 versus the control group; ##*P* < 0.01 versus LPS group

Given that NF-κB and MAPK signaling pathways are closely associated with many inflammatory diseases and play an essential role in LPS-induced lung injury in mice [30, 31], our experiment explored the anti-inflammatory of Daph on the activation of the signaling pathway. In Fig. 4a, b, we found that the protein expression of TLR4, MyD88, p-NF-κB p65, p-ERK1/2 and p-JNK significantly decreased in Daph group compared with LPS group, whereas p-p38 presented no significant difference. In addition, immunohistochemical staining was performed to detect the distribution and phosphorylation levels of NF-κB p65, ERK1/2, JNK in lung tissues. As shown in Fig. 4c, Daph significantly reduced the nucleus expression of NF-κB p65, ERK1/2, JNK compared with LPS group, suggesting the phosphorylation expression was decreased in lung tissues. At the same time, the expression of NF-κB p65, ERK1/2, JNK-positive cells in lung tissue for all groups were calculated according to immunohistochemical staining results (Fig. 4d). These results implied that Daph mediated anti-inflammatory responses might be responsible for blocking the activation of the NF-κB/MAPK signaling pathway.

### Daph reduced apoptosis and pyroptosis in LPS-induced septic lung injury

**Fig. 5:**
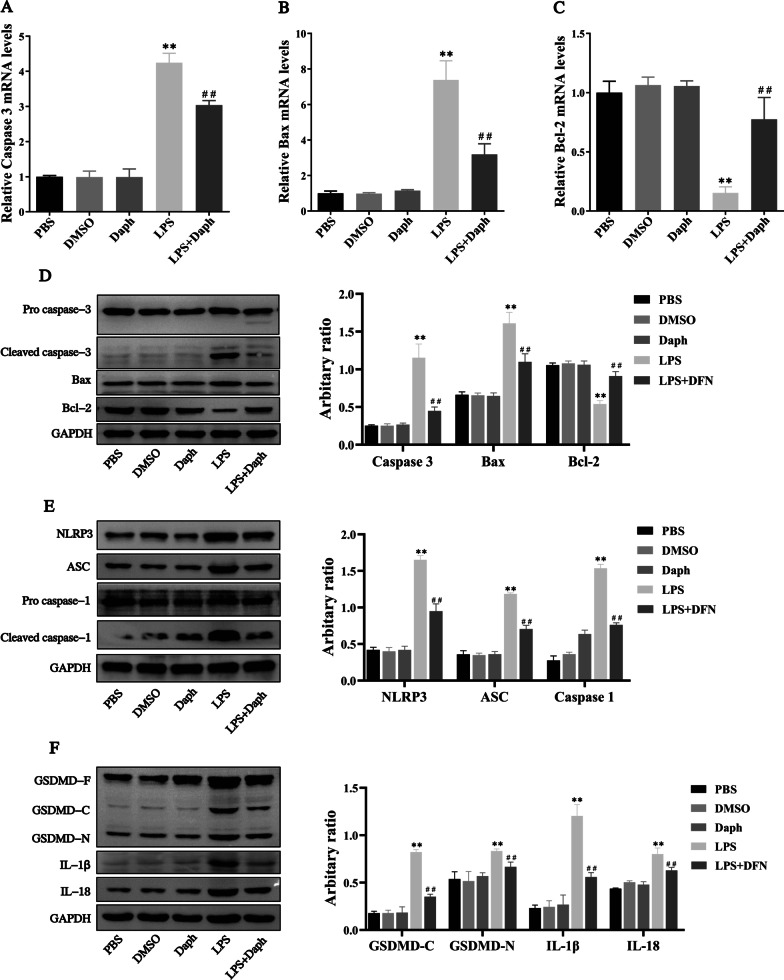
Daph treatment reduced apoptosis and pyroptosis in LPS-induced lung injury. **a-c** The mRNA expression of Caspase-3, Bax and Bcl-2 in lung tissue were detected by RT-qPCR. **d-f** The protein expression of pro caspase-3, cleaved caspase-3, Bax, Bcl-2, NLRP3, ASC, pro caspase-1, cleaved caspase-1, GSDMD, IL-1β, IL-18 were detected by Western blot analysis, and relative expression of previous protein were quantified by Image J software and GAPDH was acted as an internal control. All results were expressed as the means ± SD of three independent experiments. **P < 0.01 versus the control group; ##*P* < 0.01 versus LPS group

In the process of sepsis-related lung injury, intrarenal inflammatory infiltration in lung tissue can induce apoptosis. Therefore, we next study the effect of Daph on apoptosis of lung tissue in sepsis mice. As exhibited in Fig. 5a–c, Daph remarkedly diminished the mRNA expression of pro-apoptotic molecules caspase-3 and Bax compared with LPS group, and the mRNA expression of anti-apoptotic molecule Bcl-2 was significantly increased in Daph group. Further results from Western blot analysis consistently showed that Daph reduced the protein levels of cleaved caspase-3, Bax, and improved the protein level of Bcl-2 (Fig. 5d).

The NLRP3 is a component of the inflammasome highly expressed in various inflammatory diseases. To determine whether Daph could affect the NLRP3 inflammasome signaling pathway to improve lung injury, we measured the protein expression of NLRP3, cleaved caspase-1, ASC by Western blot. Our results showed that Daph treatment significantly reduced the protein expression of NLRP3, ASC and cleaved caspase-1 compared with LPS group, confirming that Daph could reduce NLRP3 mediated pyroptosis and protect lung tissue from LPS stimulation, and quantification of the relative protein expression levels verified these findings (Fig. 5e).

Previous study has reported that the activation cleavage of GSDMD played an important role in pyroptosis and was regarded as a pyroptosis executioner, caspase-1 regulated the cleavage and maturation of the downstream inflammatory cytokines IL-1β and IL-18 [32]. Accordingly, we measured the protein expression of GSDMD, IL-1β and IL-18 by Western blot in LPS-induced lung injury. As shown in Fig. 5f, the protein expression of GSDMD-C, GSDMD-N, IL-1β and IL-18 were significantly lower in Daph group compared with LPS group. These results suggested that LPS could induce apoptosis and pyroptosis in lung tissue and lead to lung injury with sepsis, while Daph could significantly alleviate the lung injury.

### LPS can significantly induce pro-inflammatory cytokines in MLE-12 cells

**Fig. 6:**
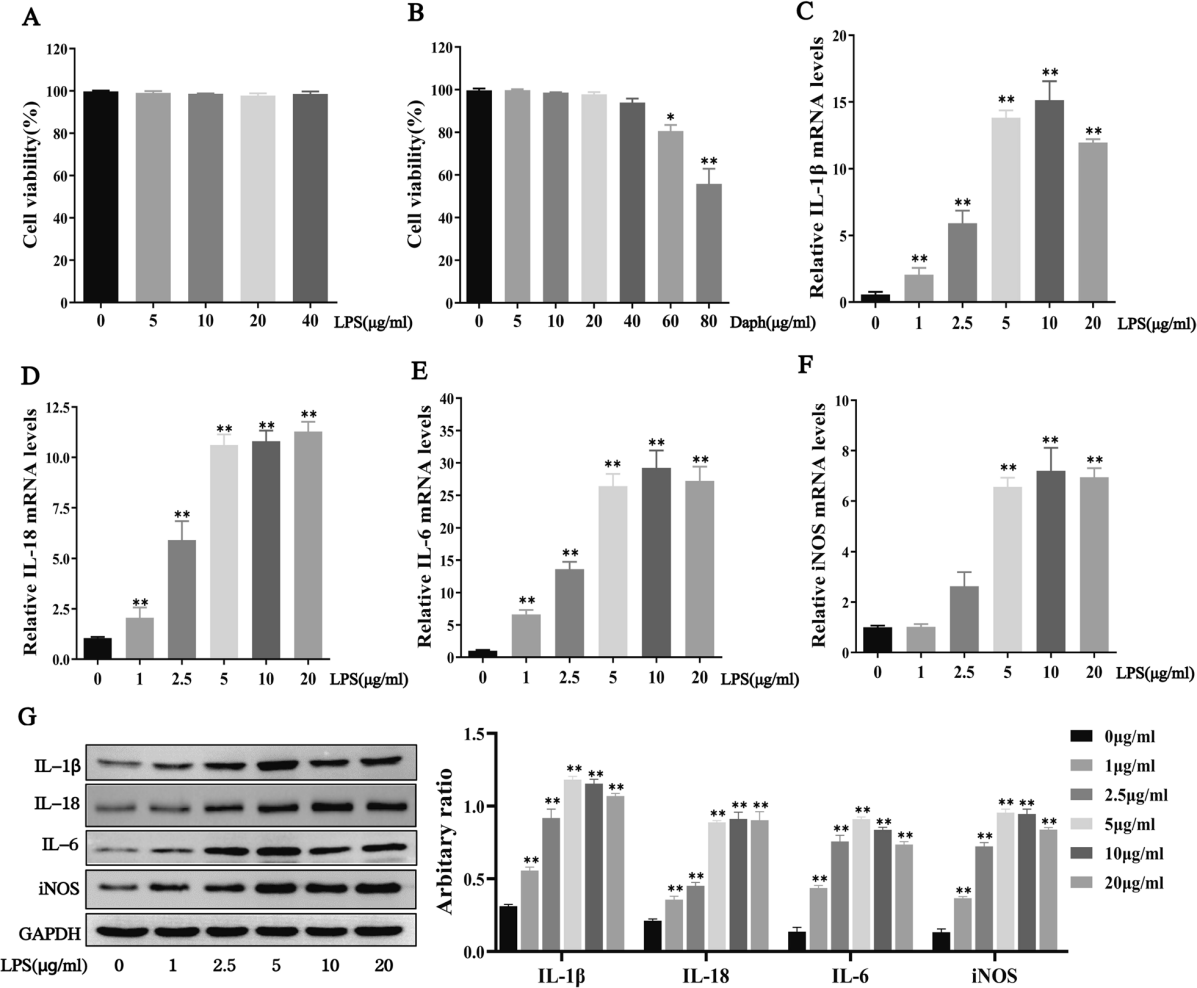
LPS could stimulate inflammatory cytokines expression in MLE-12 cells. **a-b** The viability of MLE-12 cells was analyzed by the CCK-8 assay after LPS or Daph challenge for 24 h. **c-f** The mRNA expression of IL-1β, IL-18, IL-6 and iNOS in cells were measured by RT-qPCR. **g** The protein expression of IL-1β, IL-18, IL-6 and iNOS were measured by Western blot analysis, and the relative expression of previous protein were quantified by Image J software and GAPDH was acted as an internal control. All results were expressed as the means ± SD of three independent experiments. *P < 0.05, **P < 0.01 versus 0 μg/ml LPS or 0 μg/ml Daph

Previous experimental studies found that LPS induced inflammatory cytokines expression in lung tissue of sepsis mice. Next, we wanted to explore the effect of LPS on cellular inflammatory response by constructing cells model. Firstly, MLE-12 cells were treated with LPS or Daph for 24 h at an increasing concentration, and then cell viability was determined by CCK-8 assay. In Fig. 6a, b, we found that LPS or Daph concentration in the range of 40 μg/mL had no significant effect on the viability of MLE-12 cells. As expected, LPS stimulation in MLE-12 cells caused a remarkable expression of pro-inflammatory cytokines, including IL-1β, IL-18, IL-6 and iNOS. At the concentration of 5 μg/ml after 24 h of LPS, the gene and protein levels of pro-inflammatory cytokines increased significantly (Fig. 6c–g). Thus, LPS could induce inflammation in MLE-12 cells and 5 μg/ml LPS was chosen in our following study.

### Daph attenuated inflammatory response via inhibiting TLR4-NF-κB and MAPK signaling pathways in MLE-12 cells

**Fig. 7:**
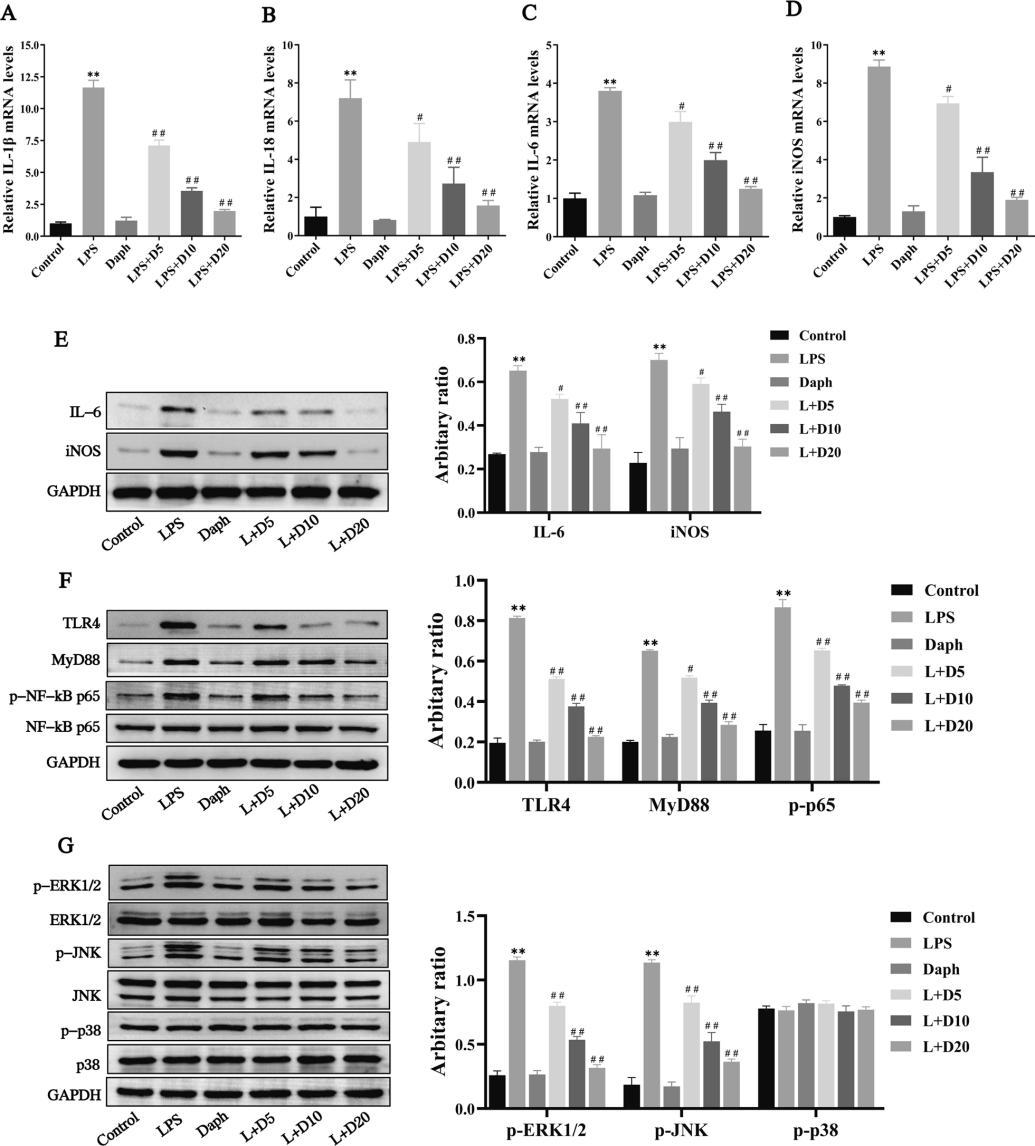
Daph treatment attenuated TLR4-NF-κB/MAPK-mediated inflammation in MLE-12 cells. **a-d** The mRNA expression of IL-1β, IL-18, IL-6 and iNOS in cells were measured by RT-qPCR. **e–g** The protein expression of IL-6, iNOS, TLR4, MyD88, NF-κB p65, p-NF-κB p65 p-ERK1/2, ERK1/2, p-JNK, JNK, p-p38, p38 in cells were measured by Western blot analysis, and relative expression of previous protein were quantified by Image J software and GAPDH was acted as an internal control. All results were expressed as the means ± SD of three independent experiments. **P < 0.01 versus the control group; #*P* < 0.05, ##*P* < 0.01 versus LPS group

Next, we would like to further study whether TLR4-NF-κB/MAPK signaling pathway affects Daph alleviating inflammatory response in MLE-12 cells. Our experimental results have shown that treatment with Daph dramatically decreased the gene and protein levels of IL-1β, IL-18, IL-6 and iNOS compared with LPS group (Fig. 7a–d), and also the protein expression of pro-inflammatory cytokines showed the same trend with mRNA (Fig. 7e). It was interesting to note that Daph with a dose of 20 μg/ml displayed more effectiveness in the reduction of pro-inflammatory cytokines. In addition, Daph treatment remarkedly inhibited the protein expression of TLR4, MyD88, p-NF-κB p65, p-ERK1/2, p-JNK in cells compared with LPS group, while p-p38 protein were not significantly difference (Fig. 7f, g). The above results manifested that Daph significantly ameliorated inflammation via inactivating TLR4-NF-κB and MAPK pathways and further inhibited the pro-inflammatory cytokines production.

### Daph treatment reduced apoptosis and pyroptosis induced by LPS in MLE-12 cells

**Fig. 8:**
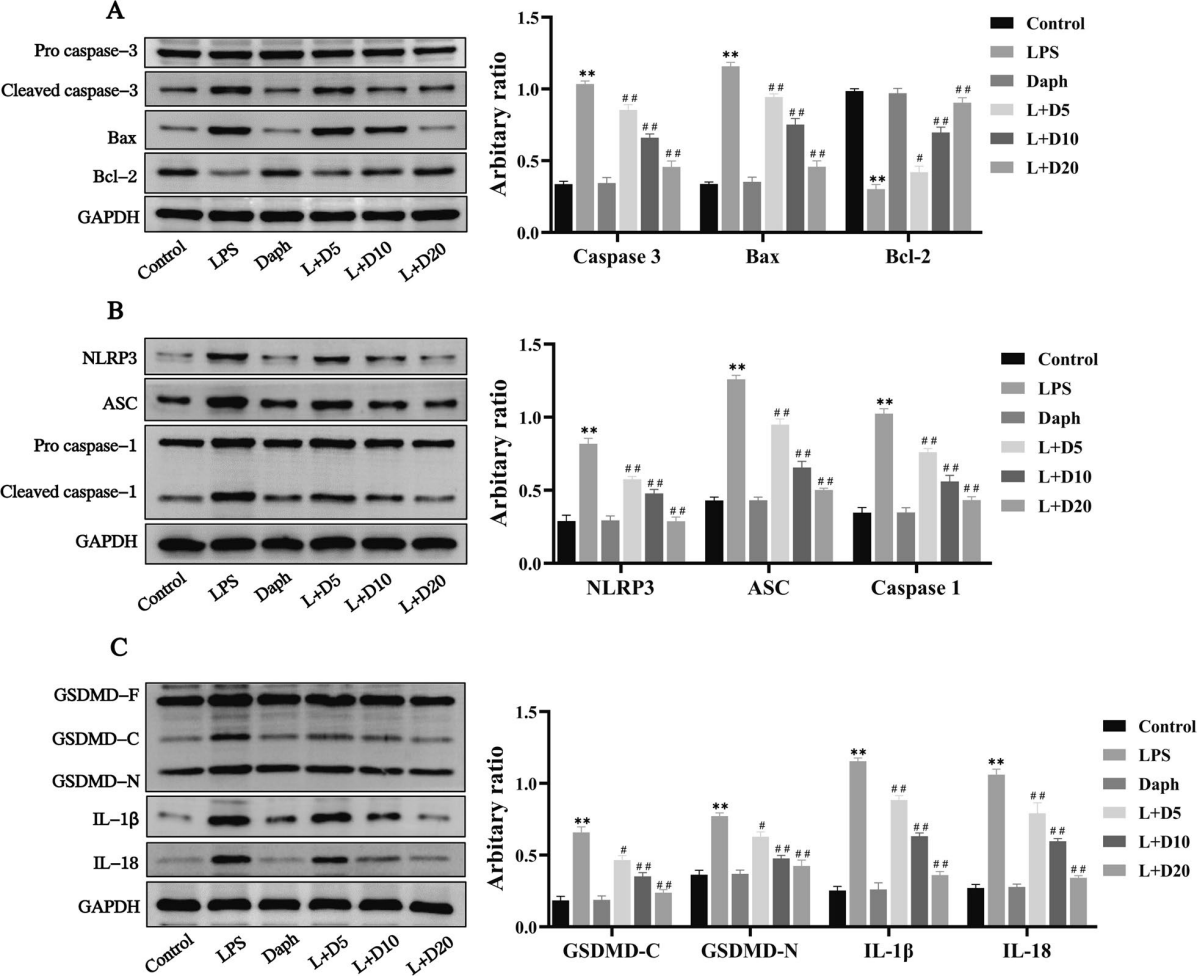
Daph reduced apoptosis and pyroptosis induced by LPS stimulation in MLE-12 cells. **a-c** The protein expression of pro caspase-3, cleaved caspase-3, Bax, Bcl-2, NLRP3, ASC, pro caspase-1, cleaved caspase-1, GSDMD, IL-1β, IL-18 were detected by Western blot analysis, and relative expression of previous protein were quantified by Image J software and GAPDH was acted as an internal control. All results were expressed as the means ± SD of three independent experiments. **P < 0.01 versus the control group; ##*P* < 0.01 versus LPS group

Previous studies have shown the critical role of apoptosis associated proteins Bcl-2, Bax and cleaved caspase-3 during the development of LPS-induced lung injury [33]. Thus, we detected the expression of Bcl-2 and Bax, as well as cleaved caspase-3 induced by LPS in MLE-12 cells. As expected in Fig. 8a, our results demonstrated that Daph significantly inhibited the expression of cleaved caspase-3 and Bax induced by LPS and reversed the inhibitory effect of LPS on the expression of Bcl-2 (Fig. 8a). Since the exposure to Daph led to a very strong reduction of NLRP3, ASC, cleaved caspase-1 in LPS-induced lung injury, we hypothesized that the observed activation of NLRP3, ASC, caspase-1 in response to Daph occurred by LPS in MLE-12 cells. To assess whether the inhibition of cytokines release by Daph was associated with decreased availability of cleaved caspase-1 or altered expression of inflammasome components, the protein expression of NLRP3, ASC, and cleaved caspase-1 were evaluated by western blot. As shown in Fig. 8b, the protein expression of NLRP3, ASC, and cleaved caspase-1 were induced by LPS and those increase were inhibited by Daph. Additionally, we also examined the protein expression of GSDMD, IL-1β and IL-18 in MLE-12 cells induced by LPS. Consistently, Daph significantly decreased the protein expression of GSDMD-C, GSDMD-N, IL-1β and IL-18 compared with LPS group (Fig. 8c). All above findings indicated that Daph might attenuate inflammatory response in MLE-12 cells through suppressing the occurrence of apoptosis and pyroptosis.

### Daph up-regulated CD38 expression in MLE-12 cells

**Fig. 9:**
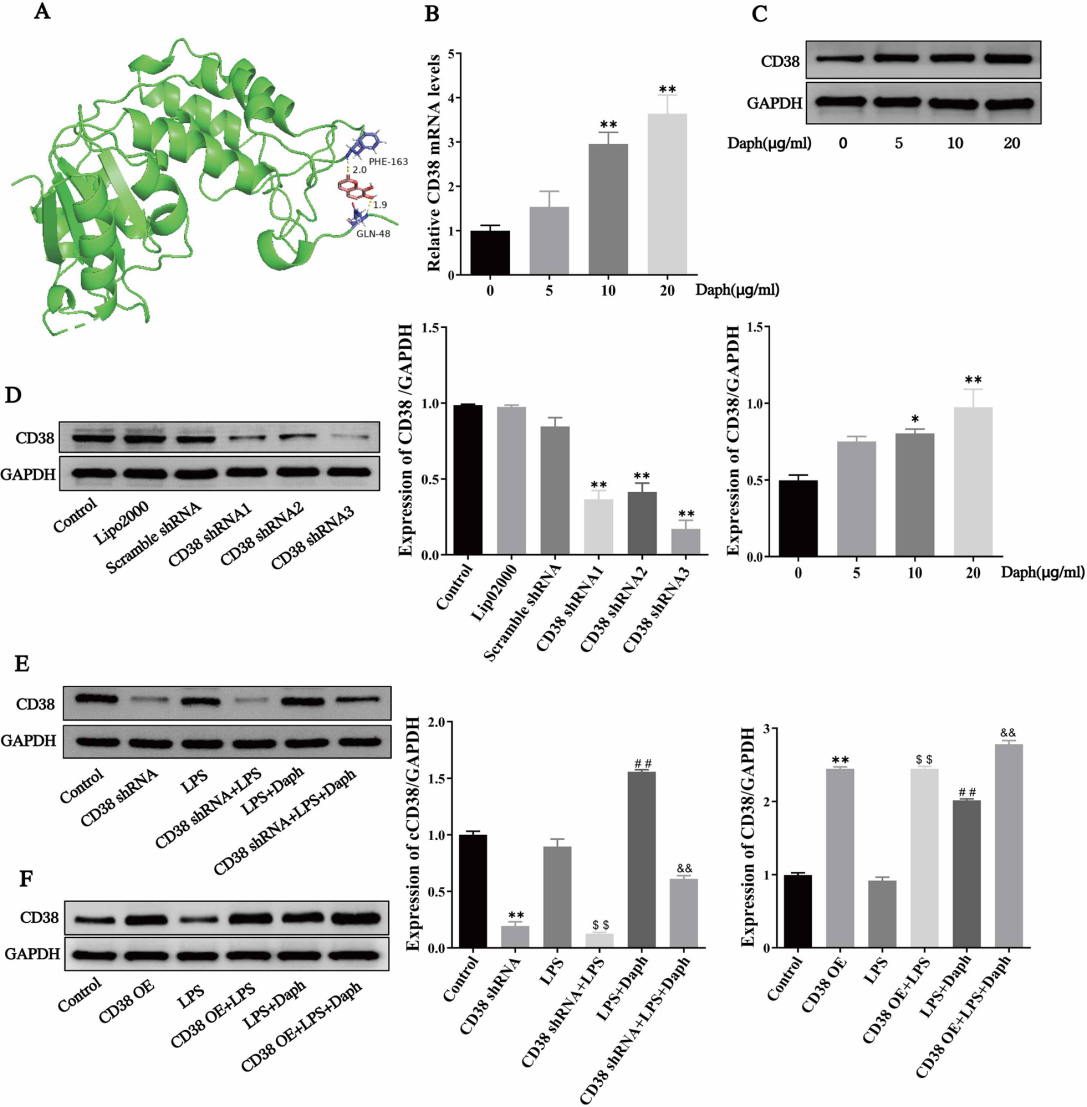
CD38 shRNA or overexpressed plasmid was successfully transfected into MLE-12 cells and Daph up-regulated CD38 expression. **a** The molecular docking diagram of Daph with CD38 molecule. **b** The mRNA expression of CD38 after Daph stimulating MLE-12 cells were measured by RT-qPCR. **c** The protein expression of CD38 in MLE-12 cells were detected by Western blot analysis, and relative expression of CD38 to GAPDH was analyzed by Image J software. **d** The protein expression of CD38 in MLE-12 cells after CD38 shRNA transfection were detected by Western blot analysis, and relative expression of CD38 to GAPDH was analyzed by Image J software. **e–f** MLE-12 cells were transfected with CD38 shRNA or overexpression plasmid for 24 h and followed by LPS and Daph treatment as indicated, the protein expression of CD38 was detected by Western blot, and relative expression of CD38 to GAPDH were analyzed by Image J software. All results were expressed as the means ± SD of three independent experiments. *P < 0.05, **P < 0.01 versus 0 μg/ml Daph or the control group; $$*P* < 0.01, ##*P* < 0.01 versus LPS group; &&*P* < 0.01 versus LPS + Daph group

Next, we intend to further explore the role of CD38 in cellular inflammation with the help of cell model. To investigate whether the anti-inflammatory effect of Daph were related to CD38. Firstly, we conducted molecular docking experiments between Daph and CD38 protein through PyMOL 2.3.0 and LigplotV 2.1 software. As shown in Fig. 9a, our results indicated that Daph has a good binding effect with CD38. In addition, MLE-12 cells were treated with different concentration gradients of Daph (5, 10, 20 μg/ mL) for 24 h as indicated. The results showed Daph could upregulate the mRNA and protein expression of CD38 in a concentration dependent manner (Fig. 9b, c). To study the role of CD38 in MLE-12 cells induced by LPS, MLE-12 cells were transfected with CD38 short hairpin RNA plasmid (CD38 shRNA) or CD38 overexpression plasmid (CD38 OE) for 24 h, followed by LPS and Daph treatment as indicated, and divided into 6 groups. Firstly, the protein expression of three sequences of CD38 were downregulated in cells transfected with CD38 shRNA compared with those transfected with scramble shRNA at 24 h after transfection, and CD38 shRNA3 plasmid was selected because of its knockdown efficiency was the best (about 80%) in MLE-12 cells (Fig. 9d). Additionally, our results showed that the knockdown or overexpression efficiency of CD38 was substantiated at the protein level using western blot analysis, and the results showed that CD38 expression in the CD38 shRNA or CD38 OE group was significantly decreased or increased compared with the control group (Fig. 9e, f). Accordingly, these results indicated that CD38 plasmid was successfully transfected into MLE-12 cells and significantly down-regulated or up-regulated CD38 expression, and further suggested that Daph could remarkedly up-regulate CD38 expression at a dose-dependent manner in MLE-12 cells.

### Knocking down CD38 aggravated inflammatory response in MLE-12 cells induced by LPS through activating TLR4-NF-κB/MAPK signaling pathway

**Fig. 10:**
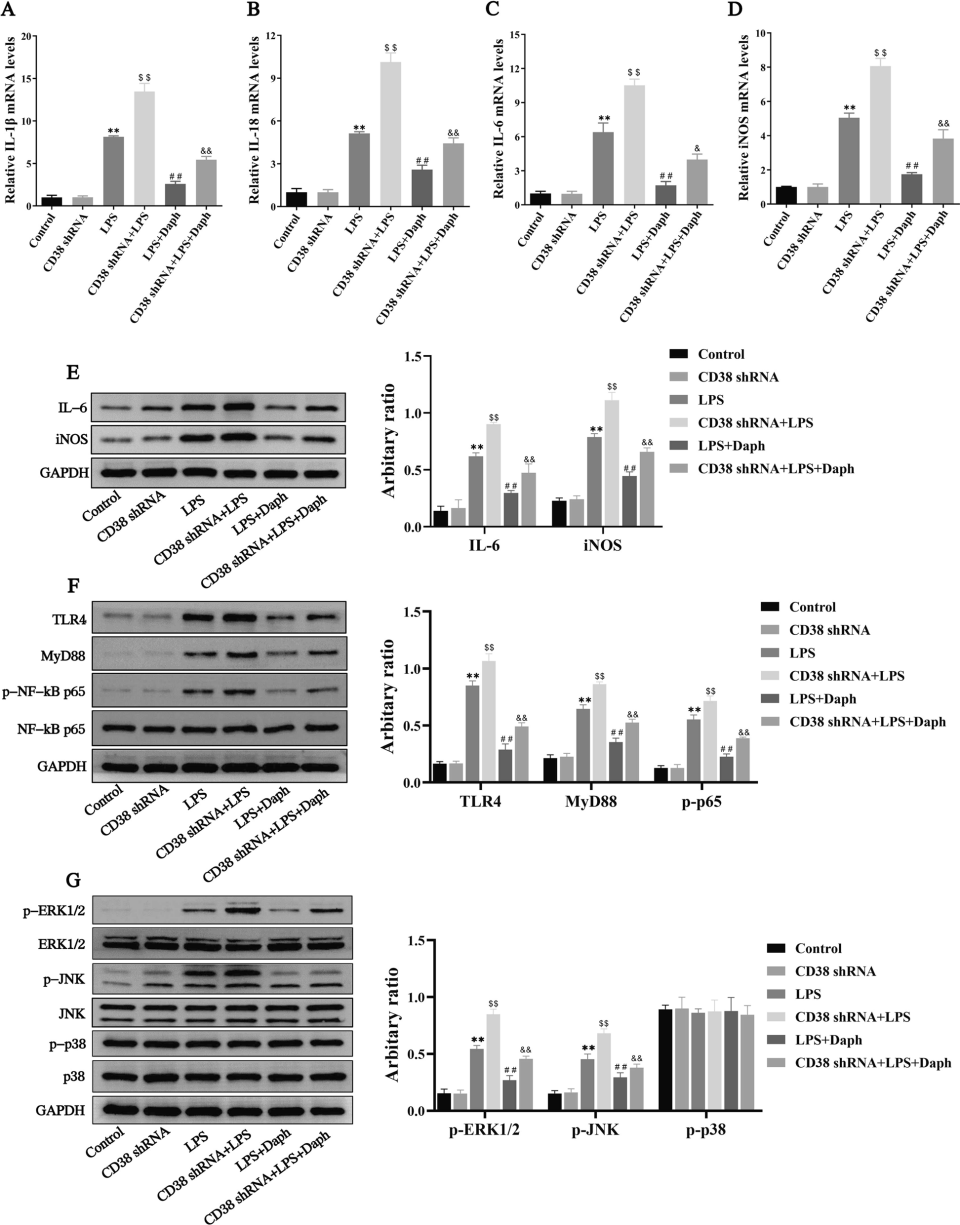
Knocking down CD38 expression intensified inflammation in LPS-induced MLE-12 cells. **a-d** The mRNA expression of inflammatory cytokines IL-1β, IL-18, IL-6 and iNOS in cells were measured by RT-qPCR. **e–g** The protein expression of IL-6, iNOS, TLR4, MyD88, NF-κB p65, p-NF-κB p65 p-ERK1/2, ERK1/2, p-JNK, JNK, p-p38, p38 in cells were measured by Western blot analysis, and relative expression of previous protein were quantified by Image J software and GAPDH was acted as an internal control. All results were expressed as the means ± SD of three independent experiments. **P < 0.01 versus the control group; $$*P* < 0.01, ##*P* < 0.01 versus LPS group; &*P* < 0.05, &&*P* < 0.01 versus LPS + Daph group

To determine whether CD38 was responsible for Daph' anti-inflammatory effects in MLE-12 cells induced by LPS. Therefore, MLE-12 cells were transfected with scramble or CD38 shRNA for 24 h, followed by LPS and Daph treatment as indicated. As shown in Fig. 10a–e, silencing CD38 significantly increased the expression of IL-1β, IL-18, IL-6 and iNOS in LPS-induced MLE-12 cells compared with LPS group. Importantly, knocking down CD38 exacerbated inflammation and attenuated the anti-inflammatory effects of Daph. Furthermore, the protein expression of TLR4, MyD88, p-NF-κB p65, p-ERK1/2 and p-JNK in the CD38 shRNA + LPS group were significantly increased compared with LPS group, expect for p-p38 protein(Fig. 10f, g). Meanwhile, CD38 knockdown significantly activated the TLR4-NF-κB/MAPK pathway to inhibit the anti-inflammatory activity of Daph (Fig. 10f, g). These findings indicated that knocking down CD38 increased inflammatory response and weakened the anti-inflammatory effect of Daph through markedly activating TLR4-NF-κB/MAPK signaling pathway in LPS-induced MLE-12 cells.

### Blockade of CD38 exacerbated the activation of apoptosis and pyroptosis induced by LPS in MLE-12 cells

**Fig. 11:**
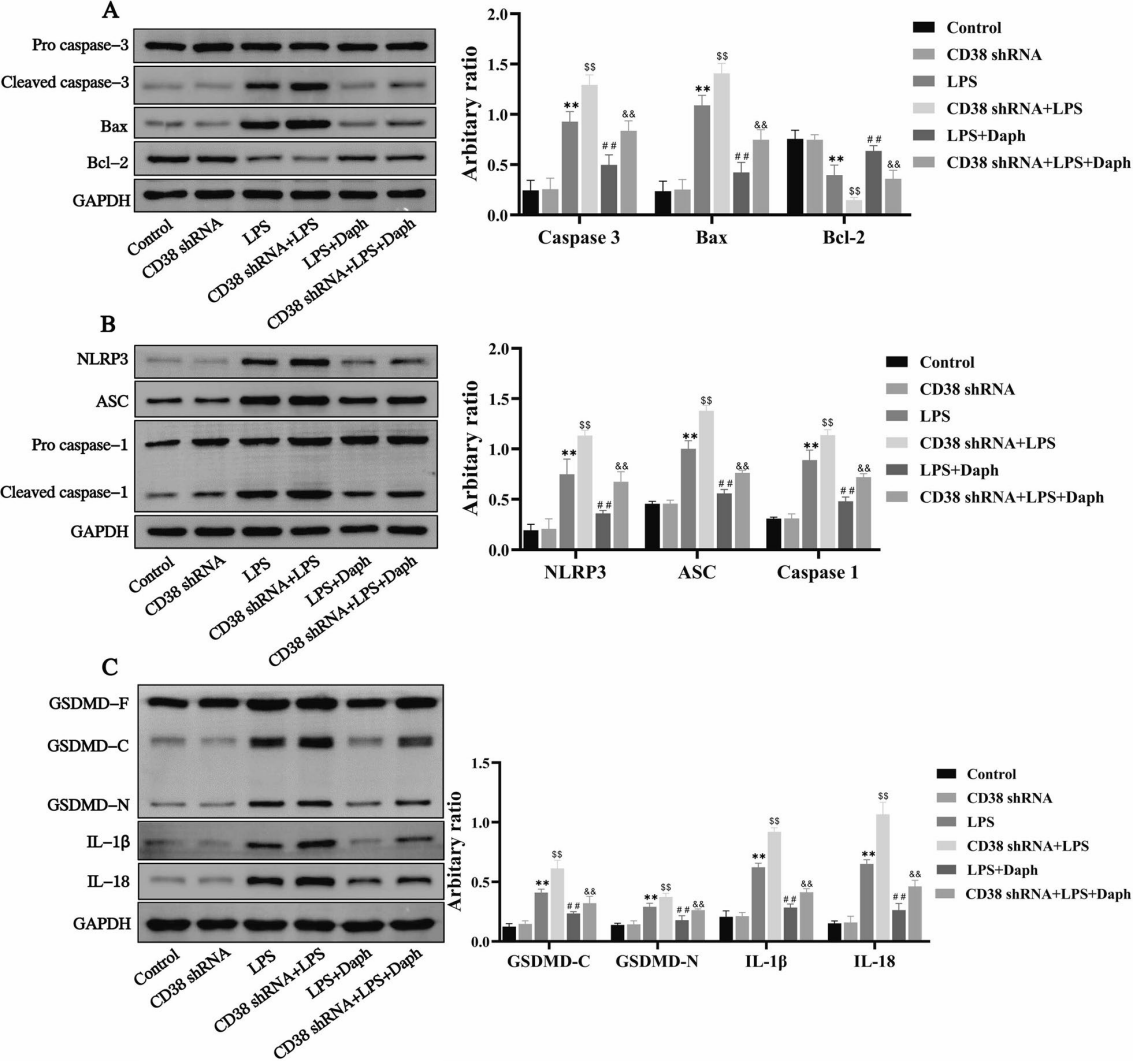
Blockade of CD38 exacerbated the activation of apoptosis and pyroptosis induced by LPS in MLE-12 cells. **a-c** The protein expression of pro caspase-3, cleaved caspase-3, Bax, Bcl-2, NLRP3, ASC, pro caspase-1, cleaved caspase-1, GSDMD, IL-1β, IL-18 were detected by Western blot analysis, and relative expression of previous protein were quantified by Image J software and GAPDH was acted as an internal control. All results were expressed as the means ± SD of three independent experiments. **P < 0.01 versus the control group; $$*P* < 0.01, ##*P* < 0.01 versus LPS group; &&*P* < 0.01 versus LPS + Daph group

To investigate the role of CD38 in Daph mediating anti-apoptotic and anti-pyroptosis effects, we inhibited the expression of CD38 by using shRNA in MLE-12 cells. Our results showed knockdown of CD38 increased cleaved capase-3, Bax expression and decreased Bcl-2 expression induced by LPS in MLE-12 cells compared with LPS group (Fig. 11a). Meanwhile, the effect of Daph on cells apoptosis was suppressed by silencing CD38. Additionally, knockdown of CD38 expression was observed to remarkedly aggravate the occurrence of LPS-induced pyroptosis, such as NLRP3, ASC, cleaved caspase-1, GSDMD-C, GSDMD-N, as well as IL-1β and IL-18 compared with LPS group, and knocking down CD38 inhibited the effect of Daph on cells pyroptosis (Fig. 11b, c). Together, these findings demonstrated that blockade of CD38 obviously promoted LPS-induced apoptosis and pyroptosis in MLE-12 cells through enhancing Caspase 3/NLRP3 activation, and significantly weakened the effects of Daph on cells apoptosis and pyroptosis.

### Overexpression of CD38 reduced inflammation in MLE-12 cells induced by LPS through inhibiting TLR4-NF-κB/MAPK signaling pathway

**Fig. 12:**
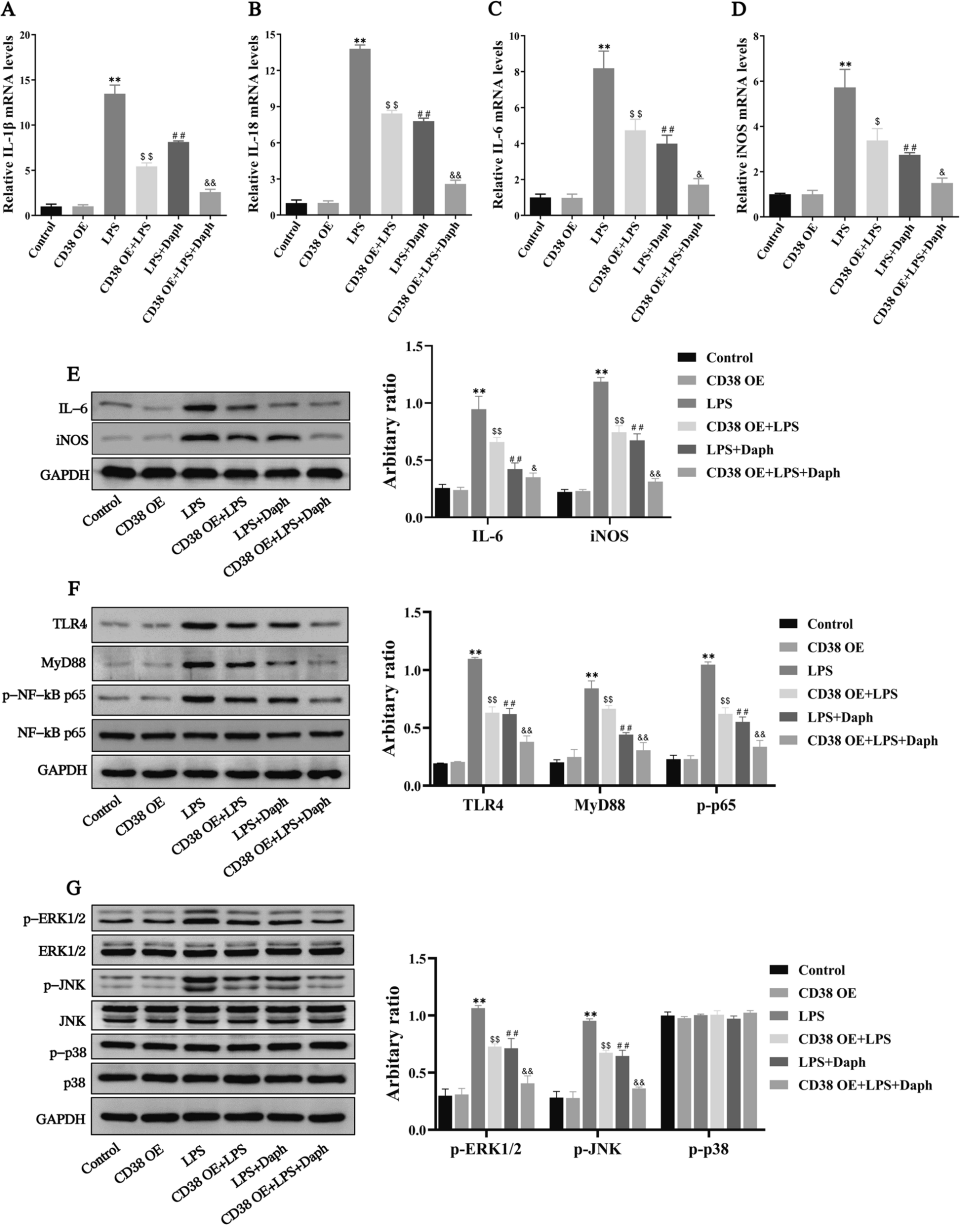
CD38 Overexpression reduced inflammation in MLE-12 cells induced by LPS. **a-d** The mRNA expression of inflammatory cytokines IL-1β, IL-18, IL-6 and iNOS in cells were measured by RT-qPCR. **e–g** The protein expression of IL-6, iNOS, TLR4, MyD88, NF-κB p65, p-NF-κB p65 p-ERK1/2, ERK1/2, p-JNK, JNK, p-p38, p38 in cells were measured by Western blot analysis, and relative expression of previous protein were quantified by Image J software and GAPDH was acted as an internal control. All results were expressed as the means ± SD of three independent experiments. **P < 0.01 versus the control group; $*P* < 0.05, $$*P* < 0.01, ##*P* < 0.01 versus LPS group; &*P* < 0.05, &&*P* < 0.01 versus LPS + Daph group

To further verify whether CD38 was responsible for Daph inhibited inflammatory cytokines expression, we established an in vitro infection model of overexpressed CD38 by transfecting LPS-treated MLE-12 cells using the prokaryotic expression vector. As shown in Fig. 12a–d, our results revealed that CD38 overexpression or Daph treatment significantly inhibited the production of IL-1β, IL-18, IL-6 and iNOS in cells compared with LPS group, and CD38 OE + LPS + Daph group exhibited lower mRNA expression of those pro-inflammatory cytokines than any other groups. At the same time, our results also indicated that the protein expression of pro-inflammatory cytokines showed the same trend with mRNA (Fig. 12e). Additionally, the protein expression of TLR4, MyD88, p-NF-κB p65, p-ERK1/2 and p-JNK in the CD38 OE + LPS group and LPS + Daph group were significantly decreased compared with LPS group, while p-p38 protein were not significantly difference (Fig. 12f, g). Interestingly, Daph significantly promoted CD38 expression to diminish activation of the TLR4-NF-κB/MAPK pathway (Fig. 12f, g). From the above results, we could know that CD38 overexpression is highly beneficial for Daph to reduce LPS-induced inflammation through TLR4-NF-κB/MAPK pathway.

### Overexpression of CD38 relieved LPS-induced apoptosis and pyroptosis in MLE-12 cells

**Fig. 13:**
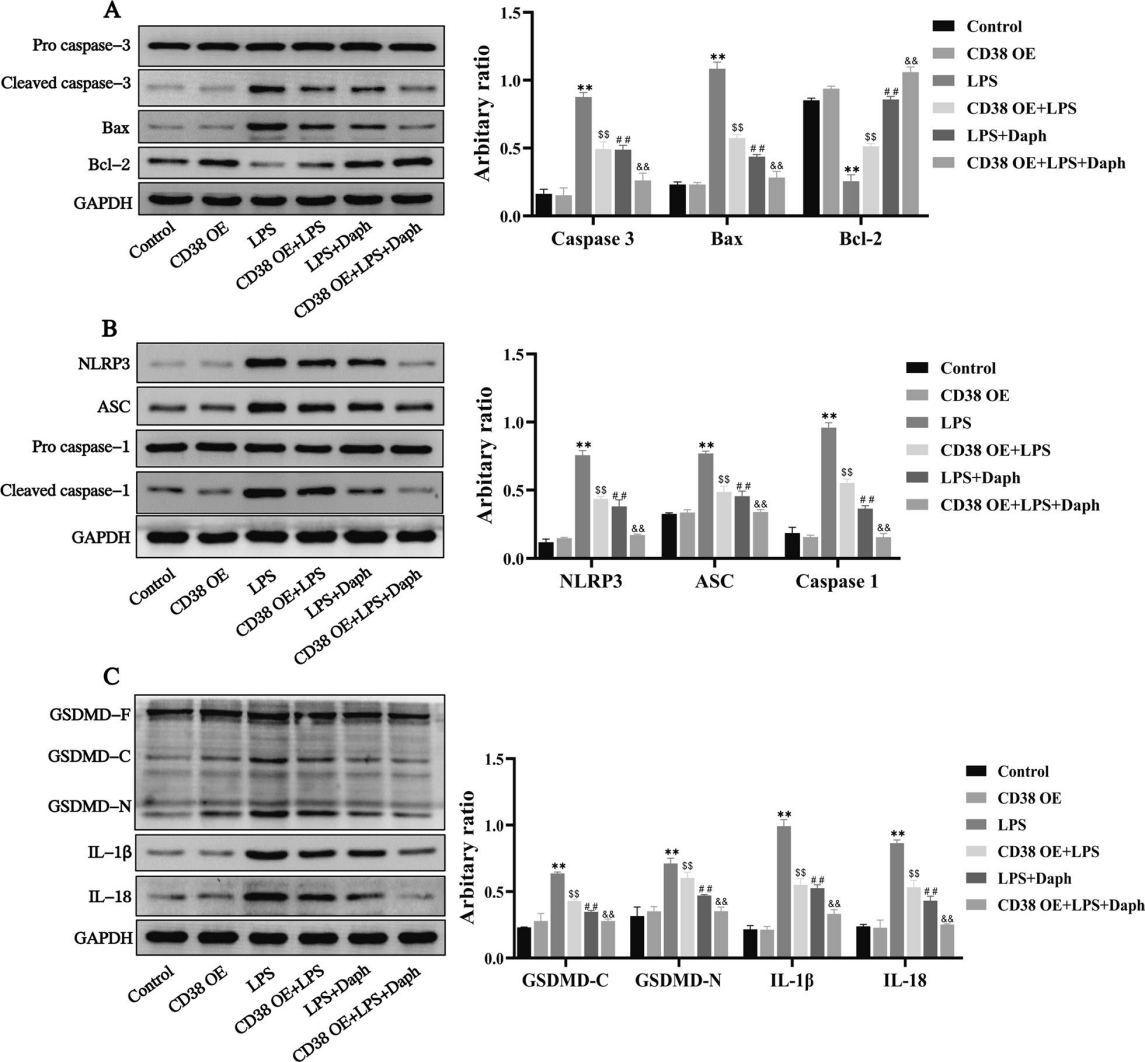
CD38 Overexpression relieved LPS-induced apoptosis and pyroptosis in MLE-12 cells. **a-c** The protein expression of pro caspase-3, cleaved caspase-3, Bax, Bcl-2, NLRP3, ASC, pro caspase-1, cleaved caspase-1, GSDMD, IL-1β, IL-18 were detected by Western blot analysis, and relative expression of previous protein were quantified by Image J software and GAPDH was acted as an internal control. All results were expressed as the means ± SD of three independent experiments. **P < 0.01 versus the control group; $$*P* < 0.01, ##*P* < 0.01 versus LPS group; &&*P* < 0.01 versus LPS + Daph group

Next, we wanted to determine the role of CD38 overexpression in LPS-induced apoptosis and pyroptosis of MLE-12 cells. As shown in Fig. 13a, CD38 overexpression and Daph treatment significantly attenuated LPS-induced the up-regulation of cleaved caspase-3, Bax and increased the expression of Bcl-2 in comparison with LPS group. Additionally, our results showed that transfection with CD38 overexpression and Daph treatment significantly reduced the protein expression of NLRP3, ASC and cleaved caspase-1 induced by LPS in cells (Fig. 13b). In accordance with the expected results, we also observed a significant decrease in the expression of GSDMD-C, GSDMD-N, as well as IL-1β and IL-18 in cells of the transfection with CD38 overexpression and Daph treatment (Fig. 13c). Taken together, these results suggested that Daph significantly increased CD38 expression to inhibit LPS-induced apoptosis and pyroptosis, thus effectively alleviating cell inflammation.

## Discussion

Sepsis caused by infection can easily progress to septic shock and life-threatening organ dysfunction, with high morbidity and mortality worldwide [34]. LPS as a principal factor causing sepsis or/and endotoxemia, leads to extensive lung injury that resulted from overwhelming inflammation [35]. Normally, the inflammatory responses can initiate tissue repair and eliminate cell injury. However, the uncontrolled inflammatory responses lead to tissue lesion, organ dysfunction, and even severe inflammatory responses syndrome with high fatality rate, such a systemic inflammatory responses syndrome (SIRS) [36]. Under LPS-induced endotoxemia, the acute lung injury(ALI) is the excessive inflammatory response in lung and can process to the deficiency of lung [37]. Therefore, any approach inhibiting inflammatory responses may potentially display the prevention and treatment of ALI. Daphnetin (Daph), a natural coumarin derivative, has been reported to exhibit various pharmacological activities, particularly its anti-inflammatory property [38, 39]. In the present study, we investigated the anti-inflammatory activity of Daph in vivo and in vitro. We found that Daph obviously protected animals from mortality, reduced the LPS-induced alveolar edema and inflammation cells infiltration in sepsis mice, and suppressed the production of pro-inflammatory factors including IL-1β, IL-18, IL-6, TNF-α and iNOS. The potential mechanisms might be related to Daph’s role in inhibiting MAPK/NF-κB/NLRP3 pathway.

In this study, we demonstrated the protective effects of Daph on LPS-induced inflammatory response in relation to CD38 in cell experiments. Knocking down CD38 aggravated inflammatory response through TLR4-NF-κB/MAPK signaling pathway and exacerbated the activation of apoptosis and pyroptosis in MLE-12 cells induced by LPS. And moreover, CD38 overexpression significantly decreased the release of pro-inflammatory cytokines in MLE-12 cells with LPS stimulated. Previous studies reported that blocking CD38 significantly inhibited LPS-induced NF-κB pathway activation and M1 polarization of macrophages, alleviating LPS-induced acute kidney injury in mice [40]. Furthermore, the lack of functional CD38 expression or the selective interference with its receptor or enzymatic activities in myeloid cells resulted in reduced production of pro-inflammatory mediators in response to LPS or to bacterial infection [41, 42]. However, CD38-deficient macrophages displayed impaired capability to phagocytose and clear Listeria monocytogenes in vitro [43]. CD38 is robustly induced during infection and the ensuing inflammation, although whether CD38 has pathogenic or regulatory effects varies depending on the diseases, immune cells, or animal models analyzed [18]. Therefore, CD38 has been clearly linked to inflammation and has been the subject of considerable study, particularly in the context of infection.

It is well known that LPS stimulates inflammatory reaction through activating a panel of intracellular signaling pathways. Among of them, MAPK and NF-κB signaling pathways are considered being in governing the production and secretion of inflammatory cytokines [44]. It has been reported that phosphorylated NF-κB p65 is highly expressed in CD38^low^ luminal cells in tissue sections, suggesting the inverse correlation of NF-κB activation and CD38 expression in luminal cells [45]. CD38 deletion also inhibited NF-κB P105 phosphorylation and alleviated pathological damage and inflammatory response in arthritic mice [46]. In IL-1β-induced astrocytes, the MAPK and NF-κB pathway regulate the increased expression of CD38 [47]. In addition, CD38 deficiency could up-regulate ERK1/2 and NF-κB pathways in sepsis mice, accompanied by the expression of inflammatory cytokines IL-1β and chemokine MCP-1 in the lung tissues, resulting in increased pathological injury in septic lung tissues [23]. In this study, we found that LPS increased TLR4 expression in turn led to the recruitment of MyD88 into the cytoplasm, increasing the nuclear translocation of ERK1/2 and JNK, stimulating the activation of the NF-κB pathway, and leading to the secretion of multiple inflammatory cytokines. And knocking down CD38 increased inflammatory response but over-expressed CD38 by CD38 overexpression plasmid or up-regulated by daphnetin can obviously inhibit inflammation. These results strongly suggested that MAPK/NF-κB signaling is involved in the occurrence and development of lung injury with sepsis, and Daph significantly increased CD38 expression to alleviate lung injury and cellular inflammatory response.

Apoptosis is induced when cells receive internal or external signals. Intrinsic apoptosis is caused by a variety of disturbances to the microenvironment, including DNA damage and endoplasmic reticulum (ER) and reactive oxygen species (ROS) stress. Several studies have explored the roles of caspase‐3, Bax and Bcl‐2 genes in the induction of apoptosis and lung injury treatment [48, 49]. Caspase 3 is the major executive caspase in apoptosis [50]. Cleavage of caspase 3 results in its activation, thus facilitating its pro-apoptotic effects [51]. Bax is a pro-apoptotic member of the Bcl-2 family of proteins, and plays a central role in mitochondria-dependent apoptosis [52]. It has been reported that hispolon suppressed Bcl-2 protein expression and increased Bax and caspase-3 protein expression, resulting in the inhibition of severe ER stress and limiting the lung injury triggered by lung cell apoptosis and lung inflammation in ALI mice induced by LPS [33]. Apoptosis of lung cells can be triggered by extracellular stimulation. Some intracellular signaling pathways, including MAPK and NF-κB, are involved in cell death. In our study, Daph significantly inhibited Cleaved-caspase 3 and Bax expression in mice and lung epithelial cells, and increased bcl-2 expression. Moreover, knocking down CD38 increased apoptosis, but CD38 overexpression obviously inhibited apoptosis induced by LPS. Therefore, Daph can up-regulate CD38 to alleviate LPS-induced apoptosis in lung injury, and further research is needed to study the possible role of apoptosis in sepsis-associated lung injury.

Pyroptosis is a programmed cell death induced by inflammation induction, and the activation of NLRP3 inflammasomes and cleavage of GSDMD play an important role in regulating pyroptosis. The classical pathway of cellular pyroptosis is initiated by inflammasomes that depend on Caspase-1. Inflammasome is a complex composed of NLRP3, adaptor protein ASC and effector protein caspase 1 precursor (Pro-caspase-1), which can recognize pathogen-associated molecular patterns (PAMPs) or damage-associated molecular pattern molecules (DAMPs). Pyroptosis is triggered primarily by NLRP3 inflammasome in a Caspase-1 dependent manner, which promotes self-cleavage of Pro-caspase 1 and activates Caspase-1. On one hand, activated Caspase-1 cleaves pre-cursors of IL-1β and IL-18 into mature IL-1β and IL-18, activates the immune system and induces inflammation [53]. Moreover, Caspase-1 leads to cleavage and polymerization of downstream GSDMD, which causes cell disintegration and perforation, providing conditions for the release of pyroptosis-related inflammatory cytokines IL-1β and IL-18, further inducing and exaggerating inflammatory response [54]. Recently, growing evidence suggests that the NLRP3 inflammasome activation is an important regulator of pyroptosis, which plays various roles in the development of lung diseases [55]. Previous studies have shown that CD38 deletion induced inflammasome-mediated Caspase-1 activation by activating NLRP3 in septic liver injury [32]. In this study, we found that Daph could regulate NLRP3/ASC/caspase-1 inflammasome complex to relieve pulmonary injury and restrain pyroptosis in MLE-12 cells. Moreover, we observed that severe cellular pyroptosis induced by LPS can be significantly reversed by CD38 overexpression, accompanied by the decrease in NLRP3 and GSDMD. Therefore, we believe that the NLRP3-GSDMD pathway regulated by CD38 plays an important role in LPS-induced MLE-12 cells and its mechanism needs to be further studied.

**Fig. 14:**
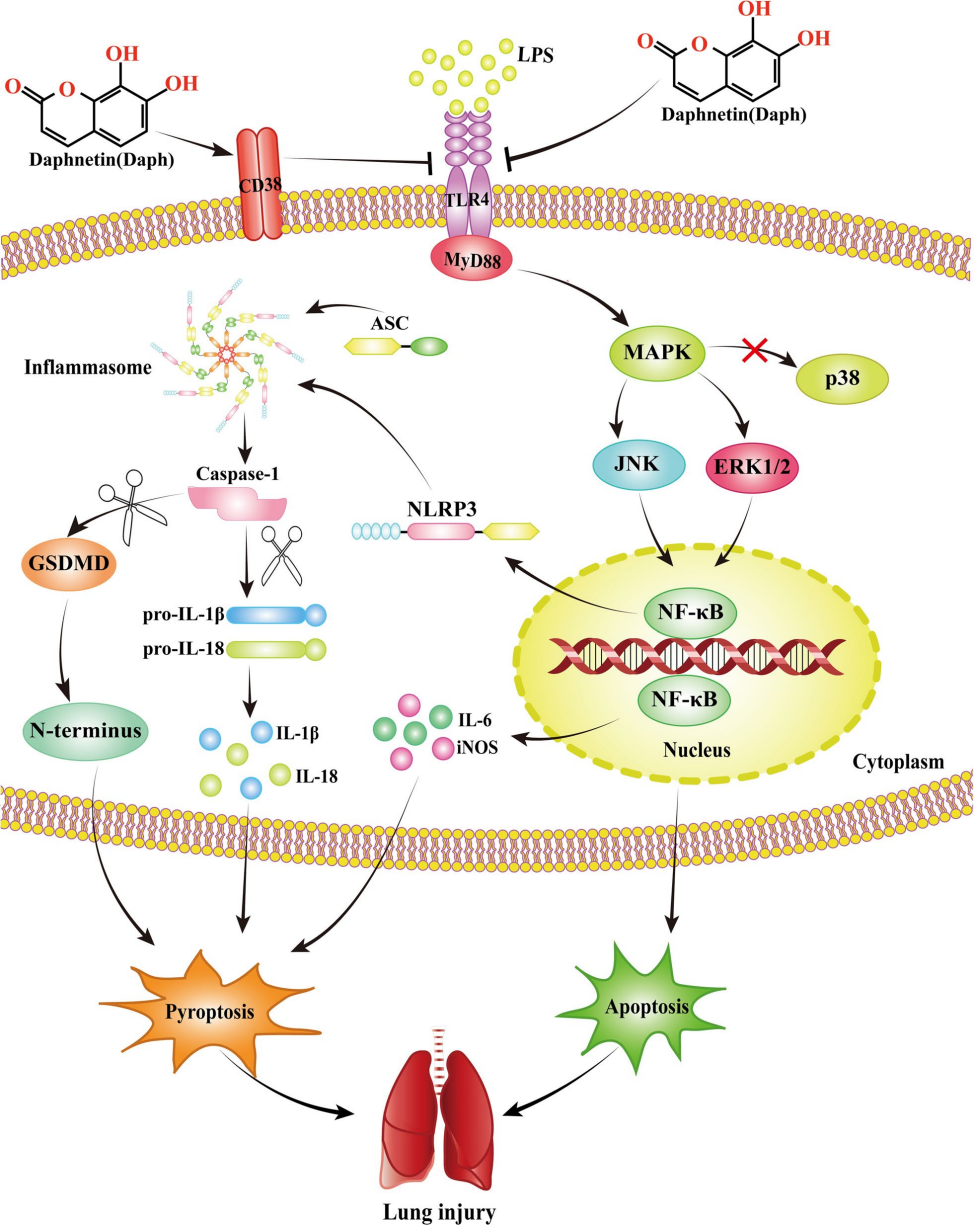
The protective effect of Daph on LPS-induced lung injury and the underlying mechanisms. Daph treatment significantly protected LPS-induced lung injury by inhibiting inflammation, apoptosis and pyroptosis, which was mediated by the up-regulation of CD38 and inhibition of MAPK/NF-κB/NLRP3 pathway

In conclusion, as illustrated in Fig. 14, we demonstrated that Daph showed anti-inflammatory activity in LPS-induced MLE-12 cells and mice via the up-regulation of CD38 and inhibition of MAPK/NF-κB/NLRP3 pathway. Additionally, three primary inflammatory pathways were CD38-dependent. According to the latest research, LPS or polymicrobial sepsis-induced mortality in CD38^−/−^ mice were markedly augmented compared with wild types, and CD38^−/−^ macrophages displayed markedly increased activation of NF-κB and NLRP3 [22]. However, the relationship between Daph and the MAPK and NLRP3 pathways in septicemic lung injury has been rarely reported. And as the above, our results have demonstrated that Daph has superior protective effects via CD38-mediated MAPK/NF-κB/NLRP3 pathway and anti-inflammatory properties. Therefore, compared to other compounds, Daph could increase CD38 expression and have better therapeutic effects, which may become a new therapy for inflammatory diseases and future studies should also address on clinical relevance of our studies.

## Conclusions

In summary, as shown in Fig. 14, Daph is capable to alleviate LPS-induced septic lung injury via the appropriate modulation of lung inflammation, apoptosis and pyroptosis. Importantly, Daph significantly upregulated CD38 expression and CD38 overexpression could remarkedly inhibited inflammatory responses in MLE-12 cells. The underlying mechanisms may be closely associated with the inactivation of the MAPK and NF-κB pathways, NLRP3 inflammasome as well as apoptosis. Daph may be proposed as one of the potential therapeutic agents to prevent septic, and the application of CD38 in Daph is beneficial to the clinical treatment of septic lung injury.

## Supplementary Information

**Additional file 1. Table S1**: Primary antibodies.

**Additional file 2. Table S2**: Sequences of the primers for real-time PCR.

**Additional file 3. Figure S3**: Original results of western blot.

## Data Availability

All data generated or analyzed during this study are included in this published article.

## Abbreviations

ALI: Acute lung injury
LPS: Lipopolysaccharide
CD38: Cluster of differentiation 38
TLR4: Toll like receptor 4
MyD88: Myeloid differentiation factor 88
MAPK: Mitogen-activated protein kinase
NF-κB: Nuclear factor κB
ERK1/2: Extracellular signal-regulated kinase 1/2
JNK: C-Jun N-terminal kinase
MCP-1: Monocyte chemotactic protein 1
CCR2: Cc chemokine receptor 2
Caspase-3: Cysteinyl aspartate specific proteinase-3
Bcl-2: B-cell lymphoma-2
Bax: Bcl2-associated X protein
iNOS: Inducible nitric oxide synthase
NLRP3: Nucleotide-binding domain (NOD)-like receptor protein 3
ASC: Apoptosis-associated speck-like protein
Caspase-1: Cysteinyl aspartate specific proteinase-1
