# Supplementary material for: Up-regulated CD38 by daphnetin alleviates lipopolysaccharide-induced lung injury via inhibiting MAPK/NF-κB/NLRP3 pathway
Source: Cell Commun Signal. 2023 Mar 30;21:66. doi: 10.1186/s12964-023-01041-3 (PMC10061746; doi:10.1186/s12964-023-01041-3)
Supplement: Supplementary file 2 — Additional file 1. Table S1: Primary antibodies. [file 12964_2023_1041_MOESM2_ESM.docx]

Supplementary Table 1. Primary Antibodies

| Name | Company | Catalog Number |
| --- | --- | --- |
| Anti-IL-1β | Cell Signaling Technology, USA | 31202S |
| Anti-IL-18 | Affinity Biosciences, China | DF6252 |
| Anti-IL-6 | Proteintech Group, USA | 66146-2-Ig |
| Anti-iNOS | Cell Signaling Technology, USA | 13120S |
| Anti-MCP-1 | Proteintech Group, USA | 66272-1-Ig |
| Anti-CCR2  Anti-Cleaved caspase-3 | Abcam, USA  Abmart, China | EPR19698  T40046F |
| Anti-BAX | Proteintech Group, USA | 50599-2-Ig |
| Anti-Bcl-2  Anti-TLR4 | Proteintech Group, USA  Cell Signaling Technology, USA | 26593-1-AP  14358S |
| Anti-MyD88 | Proteintech Group, USA | 66660-1-Ig |
| Anti- phospho-NF-kB p65 | Cell Signaling Technology, USA | 3033S |
| Anti-NF-kB p65 | Cell Signaling Technology, USA | 8242S |
| Anti-phospho-ERK1/2 | Cell Signaling Technology, USA | 4370T |
| Anti-ERK1/2 | Proteintech Group, USA | 11257-1-AP |
| Anti- phospho-p38 | Cell Signaling Technology, USA | 4511T |
| Anti-p38 | Cell Signaling Technology, USA | 8690S |
| Anti- phospho-JNK | Cell Signaling Technology, USA | 4668T |
| Anti-JNK | Cell Signaling Technology, USA | 9252T |
| Anti-NLRP3 | BOSTER,China | BA3677 |
| Anti-ASC | Affinity,China | DF6304 |
| Anti-Cleaved caspase-1 | Proteintech Group, USA | 22915-1-AP |
| Anti-GSDMD | Affinity,China | AF4012 |
| Anti-GAPDH | Proteintech Group, USA | 10494-1-1-AP |
