# Supplementary material for: Up-regulated CD38 by daphnetin alleviates lipopolysaccharide-induced lung injury via inhibiting MAPK/NF-κB/NLRP3 pathway
Source: Cell Commun Signal. 2023 Mar 30;21:66. doi: 10.1186/s12964-023-01041-3 (PMC10061746; doi:10.1186/s12964-023-01041-3)
Supplement: Supplementary file 3 — Additional file 2. Table S2: Sequences of the primers for real-time PCR. [file 12964_2023_1041_MOESM3_ESM.docx]

| Mouse Gene | Sequence |
| --- | --- |
| *F-IL-1β* | TTTTCCTCCTTGCCTCTGAT |
| *R-IL-1β* | GAGTGCTGCCTAATGTCCCC |
| *F-IL-18* | GACTCTTGCGTCAACTTCAAGG |
| *R-IL-18* | CAGGCTGTCTTTTGTCAACGA |
| *F-IL-6* | ACTTCCATCCAGTTGCCTTCTTGG |
| *R-IL-6* | TTAAGCCTCCGACTTGTGAAGTGG |
| *F-iNOS* | ATTCACAGCTACTCCGGTACG |
| *F-iNOS* | GGATCTTGACCATCAGCTTGC |
| *F-MCP-1* | TTAAAAACCTGGATCGGAACCAA |
| *R-MCP-1*  *F-CCR2*  *R-CCR2*  *F-Caspase-3*  *R-Caspase-3* | GCATTAGCTTCAGATTTACGGGT  ATCCTGCCTCCACTCTACTCCC  GGAAGAGCAGGTCAGAGATGGC  AAGATACCGGTGGAGGCTGA  AAGGGACTGGATGAACCACG |
| *F-BAX* | AGACAGGGGCCTTTTTGCTA |
| *R-BAX* | AATTCGCCGGAGACACTCG |
| *F-Bcl-2* | CTTTGAGTTCGGTGGGGTCA |
| *R-Bcl-2* | AGTTCCACAAAGGCATCCCA |
| *F-GAPDH* | GAAGGTGGTGAAGCAGGCATC |
| *R-GAPDH*  *F-CD38 shRNA1*  *R-CD38 shRNA1*  *F-CD38 shRNA2*  *R-CD38 shRNA2*  *F-CD38 shRNA3*  *R-CD38 shRNA3*  *F-CD38 overexpressed*  *R-CD38 overexpressed* | GTGGGAGTTGCTGTTGAAGTCG  GAGCATTTGTTTCCAAGAA  TTCTTGGAAACAAATGCTC  GTACTTCTGATATGAACTA  TAGTTCATATCAGAAGTAC  GTTCAAGCTCCTCCTTAAA  TTTAAGGAGGAGCTTGAAC  CACGCTGTTTTGACCTCCATAGA  ACGGCGACTACTGCACTTAT |

Supplementary Table 2. Sequences of the primers for real-time PCR
